# Lymphangioma-Like Kaposi's Sarcoma Presenting as Gangrene

**DOI:** 10.1155/2013/839618

**Published:** 2013-05-25

**Authors:** Eitan R. Friedman, Lesley Farquharson, Jessica Warsch, Ran Huo, Clara Milikowski, Margarita Llinas

**Affiliations:** University of Miami Miller School of Medicine/Jackson Memorial Hospital, 1611 NW 12th Avenue, Central 600-D, Miami, FL 33136, USA

## Abstract

Kaposi's sarcoma (KS) is a multicentric vascular neoplasm associated with the Kaposi's sarcoma-associated herpes virus (KSHV). KS can occur in immunocompromised patients as well as certain populations in Africa or in the Mediterranean. Less than 5% of KS cases can present with lymphangioma-like kaposi sarcoma (LLKS), which can occur in all KS variants. KS presents with characteristic skin lesions that appear as brown, red, blue, or purple plaques and nodules. The lesions are initially flat and if untreated will become raised. LLKS presents similarly to KS but is associated with severe lymphedema and soft tissue swelling as well as bulla-like vascular lesions. We present the case of an 85-year-old Lebanese, HIV negative, man who presented with a swollen and painful right lower extremity accompanied by necrotic lesions. Wound cultures were positive, and we began the work-up for secondarily infected gangrene. However, skin biopsy results revealed that he in fact had lymphangioma-like Kaposi sarcoma, which allowed us to shift our management. Advanced Kaposi's sarcoma can present similar to gangrene. It is important to recognize the typical skin lesions of KS and not to overlook Kaposi's sarcoma or LLKS within the differential.

## 1. Introduction

 Kaposi's sarcoma is a vascular neoplasm that develops secondary to infection with the KSHV virus. The KSHV virus is also known as the human herpes virus 8 (HHV8). KS is often seen in people of African or Mediterranean descent who have a genetic susceptibility or immunocompromised patients such as those receiving chemotherapy or in those infected with the HIV virus. Lymphangioma-like Kaposi's sarcoma is a rare subtype that presents similar to KS but is also associated with severe lymphedema and soft tissue swelling and bulla-like vascular lesions. These skin lesions can be confused with other dermatologic findings including gangrene, especially if the lesion becomes superinfected and if the disease progresses without treatment. It is important to differentiate between LLKS and gangrene so that the patient receives the correct intervention. We report a case of LLKS that was initially diagnosed as gangrene.

## 2. Case Presentation

**Figure 1:**
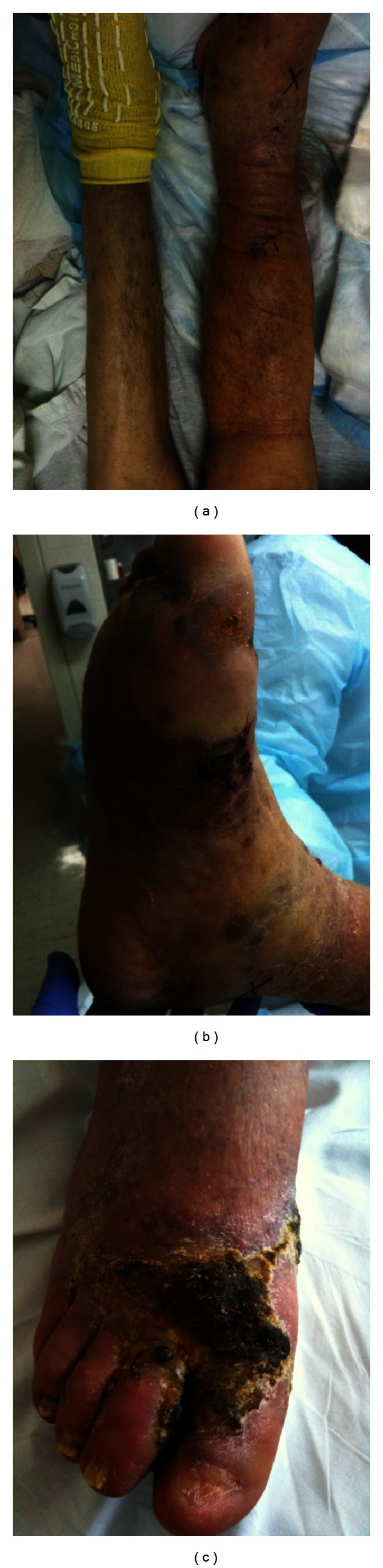
(a) Patient's right lower leg is seen here demonstrating the lymphedema compared to the opposite left leg. (b) and (c) Picture of the patient's right foot showing the blue/purple KS skin lesions as well as areas of necrosis.

We present the case of an 85-year-old Lebanese man with a history of coronary artery disease, diabetes, dyslipidemia, hypertension, and hypothyroidism who presented to our institution with a reported 2-week history of right foot edema, erythema, pain, and crusted lesions over the dorsum of the right foot. The patient was referred to our facility after receiving 2 days of IV antibiotics at a hospital in Jordan, where he had resided for the past several years. He presented to that institution after noticing red “bubbles,” which burst and crusted over with subsequent pain and swelling. He denied fevers and chills. On examination in the emergency room, the patient was noted to have weakly palpable pulses in the left lower extremity and nonpalpable pulses in the right lower extremity. His right lower extremity was edematous with blue/purple discolored lesions as well as areas of necrosis (Figure 1). The rest of his physical exam was benign with negative lymphadenopathy and lungs that were clear to auscultation, as well as negative cardiac and abdominal exams. He was initially evaluated by vascular surgery for suspected gangrene but, on arterial ultrasound with Doppler, was noted to have adequate ankle-brachial indices. Venous ultrasound with Doppler was also done to rule out deep venous thrombosis and this study was negative. Further evaluation with X-rays as well as magnetic resonance imaging (MRI) of the lower extremities was done to rule out possible osteomyelitis, given his comorbidities, and these studies were also negative. After wound cultures were done, the patient was started on broad-spectrum antibiotics for suspected soft tissue infection. Punch biopsy was also done to rule out fungal or parasite infection. After 4 days with no clinical improvement, he was taken to the operating room for debridement. Pathology from that debridement revealed Kaposi's sarcoma, lymphangioma type with positive human herpes virus-8 (HHV8) immunostain. The biopsy, which was done prior to surgery, also confirmed this diagnosis. Multiple environmental pathogens, as well as *Enterococcus faecalis*, were noted on wound culture and as such, the patient completed 2 weeks of ampicillin/sulbactam.

 Given the diagnosis of Kaposi's sarcoma, HIV testing was done, which was negative, and so no other tests for immunosuppression were performed. The patient also underwent computed tomography (CT) scans of his chest, abdomen, and pelvis to evaluate for metastatic disease. These studies were done without contrast as the patient developed acute kidney injury during his hospitalization, likely as a consequence of supratherapeutic vancomycin. Lastly, the patient received upper and lower endoscopies, both of which were negative for lesions suspicious for malignancy. As there was no evidence of metastases on this evaluation, the decision was made to provide local therapy only, under direction from dermatology. Unfortunately, the patient was lost to followup after discharge.

## 3. Discussion

Kaposi's Sarcoma is a multicentric vascular neoplasm-associated with the Kaposi's sarcoma associated herpes virus. The KSHV virus is also known as the human herpes virus 8. Studies have shown that KSHV-infected cells of vascular endothelial origin secrete cytokines, chemokines, and growth factors that allow for angiogenesis and further cancer pathogenesis [1]. The KSHV viral genome encodes several viral genes. One of the viral genes encodes for a constitutively active intramembranous viral G-protein coupled receptor (vGPCR). This vGPCR is present in only a small number of infected cells and causes a signaling cascade which allows for the downstream transcription of genes which encode for cytokines, chemokines, and growth factors which are ultimately secreted from the cell and have paracrine effects on neighboring cells causing further cell proliferation and cancer growth [2].

There are four types of KS seen clinically. These include classic variant KS, endemic KS, transplant-associated KS, and AIDS-associated KS. Classic variant KS is slow growing, often limited to the skin, and often found in people of Mediterranean descent. Endemic KS is found in Africa in HIV negative patients. This form is aggressive and can affect children. Transplant-associated KS is seen in patients who are on immunosuppressive therapy. The last type is AIDS-associated KS, which can develop in HIV positive patients [3]. It is unknown why people of Mediterranean descent or of African descent are susceptible to the KSHV; however, it is known that HIV positive patients and patients who are on chronic immunosuppressive drugs have a compromised immune system which can make them susceptible to the KSHV and subsequently to the development of KS. Thus, it can be deduced that the classic and endemic variants are caused by genetic susceptibilities to the KSHV. 

Our patient is of Mediterranean descent and is HIV negative, and the classic variant of KS in these patients tends to be more indolent in nature, these patients are often elderly as was our patients and occurs in men more often than women in a 15 to 1 ratio. The cancer often begins in the hands or feet and progresses towards the body, potentially taking several years to decades before the cancer metastasizes to the viscera [4]. KS most often metastasizes to lymph nodes, the gastrointestinal tract, and to the lungs [5]. The lesions begin as multiple firm reddish/brown or purple/blue plaques and nodules [4]. The lesions are initially flat and if left untreated will become confluent and raised. Although of Mediterranean descent, our patient had a rare type of KS known as lymphangioma-like KS, which can occur in each of the four KS variants and is less than 5% of all KS cases [6]. These patients usually present similar to KS, with blue/purple solid lesions; however, our patient also had severe lymphedema and soft tissue swelling as well as bulla-like vascular lesions characteristic of LLKS [7]. Bulla-like lesions are the most common clinical feature seen in LLKS. Also, LLKS usually presents in the lower extremities, as was the case in our patient [8].

**Figure 2:**
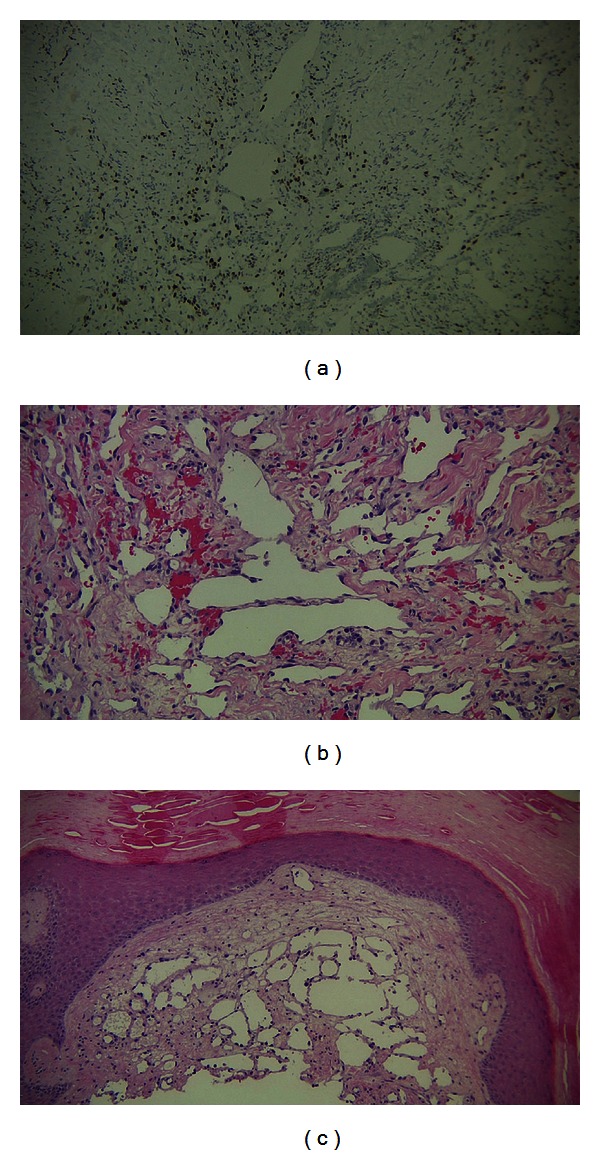
(a) Patient's skin biopsy showing immunohistochemical stain for HHV8. There is a strong nuclear reactivity in the endothelial cells as well as some of the stromal spindle cell component. (b) and (c) H and E staining of skin biopsy from the patient. Within the dermis are markedly dilated vascular spaces lined by prominent endothelial cells. Within the stroma are few spindle cells without apparent atypia. This represents the lymphangioma-like variant of Kaposi sarcoma.

The diagnosis of LLKS can be made based on clinical presentation and on histological examination of the lesions. LLKS is typified histologically by lymphangioma-like spaces. Often these patients also histologically show patterns associated with the other forms of KS. These patterns include spindle and endothelial cell proliferation, red blood cell extravasation, hemosiderin-laden macrophages, and other signs of an inflammatory reaction [5]. In addition, biopsy samples can be stained for immunohistochemistry with anti-HHV8 antibodies in order to detect the KSHV in the tumors themselves, which can be detected in all four KS variants.  Figure 2 shows the staining that was performed on our patient's biopsy sample.  Figure 2(a) shows immunohistochemical staining for HHV8, and Figures 2(b) and 2(c) show the classic lymphangioma-like spaces.

Treatment of KS includes simple excision of a single lesion and a combination of surgery, radiation, and chemotherapy for multiple and metastasized KS. 

Herein we describe a patient with an extremity lesion that was initially believed to be a necrotic gangrenous foot. Cultures from his left foot biopsy from one of his lesions grew Gram-positive cocci and Gram-negative rods. We began the patient on systemic broad-spectrum antibiotics to treat his superinfected gangrenous foot. It was only after a biopsy was attained and histological and immunohistochemical examination was performed that we discovered that the patient had LLKS and it was at that time when the oncology team was able to begin management with chemotherapy, radiation, and surgery. It is important to recognize the typical skin lesions of KS and not to overlook Kaposi's sarcoma within the differential of a patient who presented as ours did, especially in a patient who is of Mediterranean descent, elderly, and HIV negative.
